# Generation of three-dimensional multiple spheroid model of olfactory ensheathing cells using floating liquid marbles

**DOI:** 10.1038/srep15083

**Published:** 2015-10-14

**Authors:** Raja K. Vadivelu, Chin H. Ooi, Rebecca-Qing Yao, Johana Tello Velasquez, Erika Pastrana, Javier Diaz-Nido, Filip Lim, Jenny A. K. Ekberg, Nam-Trung Nguyen, James A. St John

**Affiliations:** 1 Eskitis Institute for Drug Discovery, Griffith University, Brisbane, QLD 4111, Australia; 2 QLD Micro- and Nanotechnology Centre, Griffith University, 4111, Australia; 3 School of Biomedical Sciences, Queensland University of Technology, Brisbane, 4000, QLD, Australia; 4 Nature Communications, New York, USA; 5 Centro de Biología Molecular Severo Ochoa & CIBERER, Madrid, Spain; 6 Universidad Autónoma de Madrid, Madrid, Spain

## Abstract

We describe a novel protocol for three-dimensional culturing of olfactory ensheathing cells (OECs), which can be used to understand how OECs interact with other cells in three dimensions. Transplantation of OECs is being trialled for repair of the paralysed spinal cord, with promising but variable results and thus the therapy needs improving. To date, studies of OEC behaviour in a multicellular environment have been hampered by the lack of suitable three-dimensional cell culture models. Here, we exploit the floating liquid marble, a liquid droplet coated with hydrophobic powder and placed on a liquid bath. The presence of the liquid bath increases the humidity and minimises the effect of evaporation. Floating liquid marbles allow the OECs to freely associate and interact to produce OEC spheroids with uniform shapes and sizes. In contrast, a sessile liquid marble on a solid surface suffers from evaporation and the cells aggregate with irregular shapes. We used floating liquid marbles to co-culture OECs with Schwann cells and astrocytes which formed natural structures without the confines of gels or bounding layers. This protocol can be used to determine how OECs and other cell types associate and interact while forming complex cell structures.

Spinal cord injury causes irreversible axonal damage and neuronal death, resulting in permanent deficits of sensory and autonomic function that lead to chronic paralysis. One strategy to repair the injured spinal cord is to transplant olfactory ensheathing cells (OECS) to provide a bridge over the injury site and through which regenerating axons can grow. OECs are glial cells of the olfactory nervous system which exhibit unique growth-promoting properties that can dramatically increase survival and axonal regeneration of central nervous system neurons^1^. OECs are known to promote axon growth^2^, provide neuroprotection^3^, remyelination^4^ and phagocytose neuronal debris^5^,^6^. Transplantation of OECs to an injured spinal tract has recently been successful in re-establishing functional, connections in a human who had a completely severed spinal cord. The method is safe and feasible^7^,^8^ but the results are variable with some studies showing little functional improvement^9^. The outcomes of OEC-based therapeutic approaches are often confounded by poor post-transplantation survival and the integration of OECs into the spinal cord tissue due to the complexity of spinal cord injury^10^.

To improve OEC transplantation therapies, we need to further understand the biology of OECs and how they interact with other cells types. In particular, understanding the behaviour of OECs in a complex three-dimensional (3D) culture model will help to develop new approaches for transplanting cells into the injured spinal cord. A three-dimensional culture system more accurately mimics the *in vivo* complexity as it provides the multilayer cell-cell and cell-matrix interactions within a multilayer system. Current 3D approaches of OEC cultures are mainly based on 3D extra cellular matrices (ECM) such as alginates^11^,^12^ and collagen^13^,^14^. However these scaffold-based 3D OEC cultures cannot sufficiently model the morphological features and physiological functions of the *in vivo* micromilieu as the application of exogenous scaffolds or ECM based materials may obstruct cell-cell interactions and the assembly of native ECM. In contrast, a scaffold-free 3D culture system allows suspended cells to self-assemble to form multicellular spheroids^15^. Self-assembly of spheroids results in uniform size geometry and structural properties that are optimal for drug screening and elucidating cell-cell interactions^16^. Thus, multi-cellular spheroids may provide novel insights into the role of OECs that could be used to enhance their therapeutic potential for repairing spinal cord injury.

Liquid marbles (LM) are a form of 3D bioreactor that have previously been shown to support the growth of living microorganisms^17^, tumour spheroids^18^, fibroblasts^19^, and red blood cells^20^. LMs are formed by enveloping a drop of liquid with hydrophobic powder particles to form an elastic hydrophobic shell with fine pores. The coating material acts as a confined space which is non-adhesive and allows the cells to freely interact with each other^21^. Sessile LMs which rest on a hard surface have some disadvantages as they are more difficult to handle and are subject to undesirably high evaporation. Floating LMs have very interesting properties that influence their behaviour. An air layer exists between the marble and the fluid surface^22^ which influences the marble’s movement; marbles have self-propelling motion due to evaporation^23^ and the size of the marble influences the deformation of the marble^24^. We considered that these various characteristics of floating LMs were amenable to culturing cells in three dimensions. We have now developed a microfluidic bioreactor platform in which LMs float in cell culture medium and can be used to generate multiple uniform-sized OEC spheroids. We show that floating LMs produce superior spheroids to sessile LMs.

An additional advantage of floating LMs is the potential to co-culture different cell types in three dimensions to better understand cellular organization and interactions. To demonstrate the efficacy of floating LMs for co-culture, we examined the interaction of OECs with Schwann cells and with astrocytes and found that OECs preferentially enveloped the other cells. Such cell interactions by OECs have not been previously reported and may provide crucial insights how these cells behave after transplantation to injured nerve tracts, ultimately resulting in increased therapeutic potential.

## Results

### Production of floating liquid marbles

**Figure 1:**
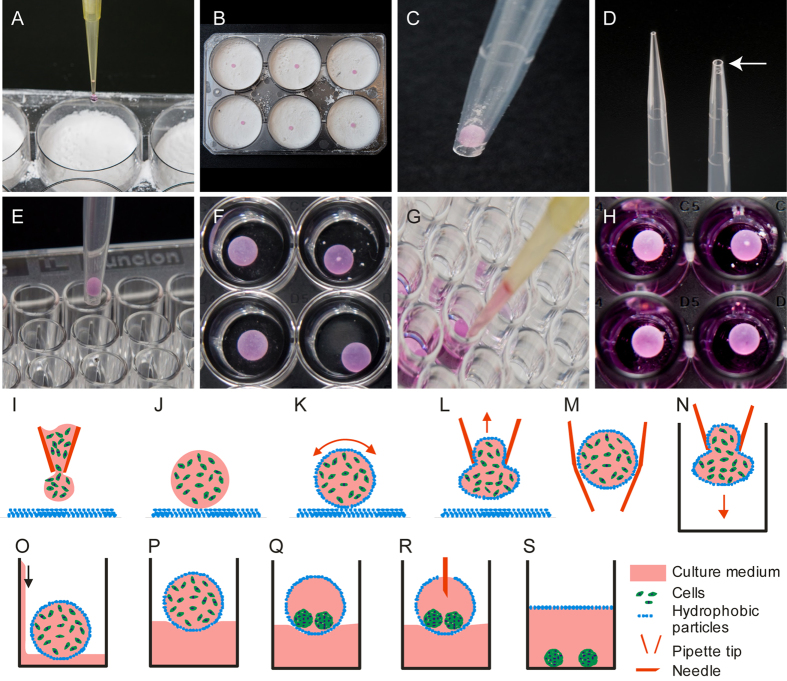
Preparation of floating liquid marble containing cells. (**A**,**B**) Drop a predetermined volume of cells containing media on the polytetrafluoroethylene (PTFE) powder in 6-well plates. (**C**,**D**) Marble is picked up and transferred using a p1000 pipette (white arrow indicates the cut p1000 pipette tip). (**E**,**F**) Transfer the marble to a 96-well plate and (**G**,**H**) float the marbles with media. Process flow for using floating liquid marble. (**I**,**J**) A droplet of cells is deposited on the PTFE powder bed and (**K**) then coated with PTFE by rotating the marble with circular movements. (**L**–**N**) The marble is picked up and transferred using a pipette tip and (**O**,**P**) is then floated by dispensing media against the wall of the well. (**Q**) After incubation, the cells form spheroids inside the marbles. (**R**,**S**) Marbles can be broken with needles to release the spheroids which then sink to the bottom of the well.

To create the LM, the surface of liquid droplet was coated with hydrophobic particles which produces a coating those envelopes the liquid droplet. We used polytetrafluoroethylene (PTFE) powder (average particle size of 1 μm) produced with a maximum grind of 2.0 NPIRI (Sigma-Aldrich, product number 430935). A single droplet containing a predetermined number of OECs in a preferred volume of between 10 to 50 μL was dispensed onto the powder bed (Fig. 1B,I,J). The plate was gently rotated in a circular motion so the powder particles covered the surface of the liquid droplet and formed the liquid marble (Fig. 1K). Importantly, it was crucial to use clean PTFE powder because previously used PTFE powder tends to cause aggregation of particles. The marbles were subsequently picked up using 1000 μL pipette tips which were cut at the edge to accommodate the diameter of the marble (Fig. 1C,D). The marbles were sucked up into the pipette tip and then dispensed into a 96-well plate (Fig. 1E,F,L–N). The approximate diameter of the opening pipette tip is slightly less than the marble diameter (Fig. 1M) which creates a friction fit to grip the marble inside the tip. To float the marbles, 100 μL of media was added from the margin of the well to slowly bathe the marble (Fig. 1G,H,O,P). Direct liquid contact disrupts the hydrophobicity of the coated PTFE powders and breaks the marbles. After incubation at 37 °C, the LM content can be monitored using optical–fluorescent microscopy to examine the cell growth and spheroid formation (Fig. 1Q). The fine coating of hydrophobic powders with particle size of 1 μm allows optical access to the content of the marble. Therefore, this protocol is well suited to studies of the assembly of cells with different fluorescent labels by using time-lapse microscopy. Finally marbles can subsequently be broken by puncturing with a needle (Fig. 1R,S) to obtain spheroids.

### Optimizing the marble size for multiple spheroid generation

**Figure 2:**
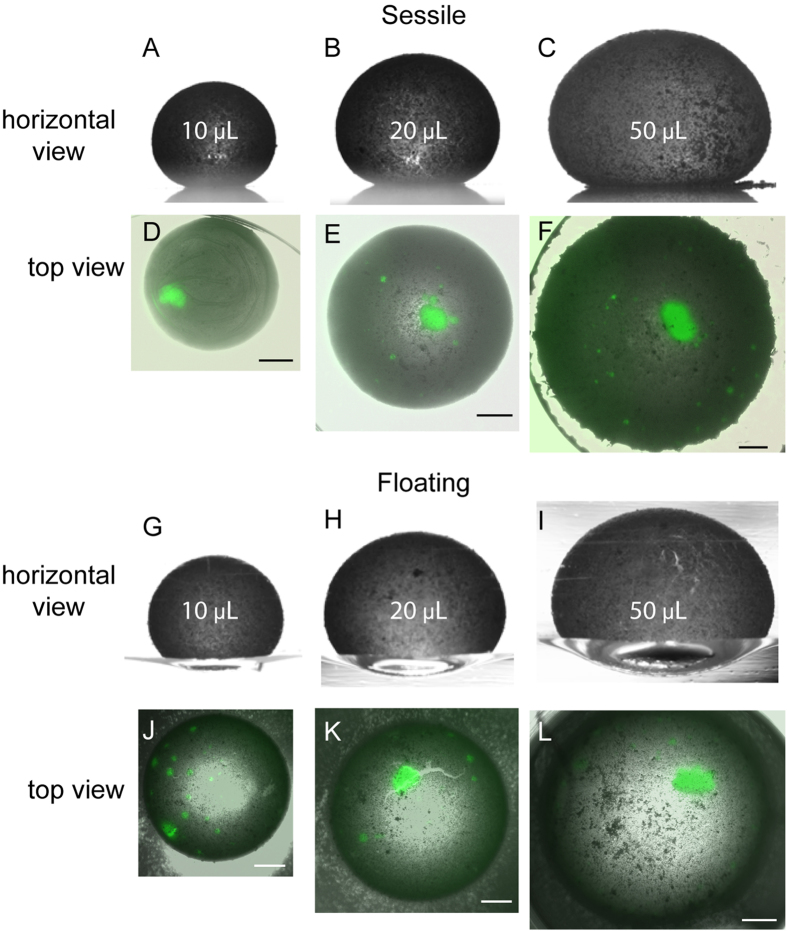
Effect of marble volume on spheroid formation. (**A**–**C**) Horizontal micrographs of sessile liquid marbles show the flat bottom of 20 μL and 50 μL marbles. (**D**,**E**) Bright field and fluorescent merged microscopic images show the spheroids inside the liquid marbles. (**G**–**I**) Horizontal view of floating liquid marbles showing the curved lower surface. (**J**) 10 μL floating marbles produced multiple spheroids, whereas (**K**,**L**) 20 μL and 50 μL marbles produced fewer spheroids. Scale bar is 500 μm.

To determine the ideal volume of LM for multiple OEC spheroid production, we prepared LMs with OECs at a fixed density of 500 cells/μL in volumes of 10 μL, 20 μL, and 50 μL. The volume-dependent spheroid formation was performed in sessile and floating conditions as shown in Fig. 2. The horizontal profiles of the liquid marbles were viewed by bright field micrographs. While the 10 μL sessile LMs were spherical (Fig. 2A), sessile LMs with a volume of 20 μL and 50 μL were not spherical and had a flat bottom where they rested on the surface of the dish (Fig. 2B,C). Fluorescent microscopy was used to view the cells within the sessile LMs which showed that the cells underwent sedimentation and formed an irregular shaped mass of cells (Fig. 2D–F). In contrast, floating LMs retained their spherical shape at the bottom surface (Fig. 2G–I), and cells aggregated to also produce masses of cells (Fig. 2K,L). Interestingly, the 10 μL floating LMs generated numerous small spheroids (Fig. 2J) while the large volume floating LMs tended to produce at least one large mass of cells (Fig. 2K,L). As the 10 μL floating LMs formed multiple spheroids, we compared them to the mass of tissue formed within the sessile 10 μL LMs. Despite having a spherical bottom, the sessile LM did not form multiple spheroids but instead tended to form a large irregular shaped mass of tissue (Fig. 2A). These results indicated that the 10 μL floating LMs were optimal for producing multiple spheroids through free association of cells rather than through sedimentation of cells.

### Optimizing the cell density for multiple spheroid generation

**Figure 3:**
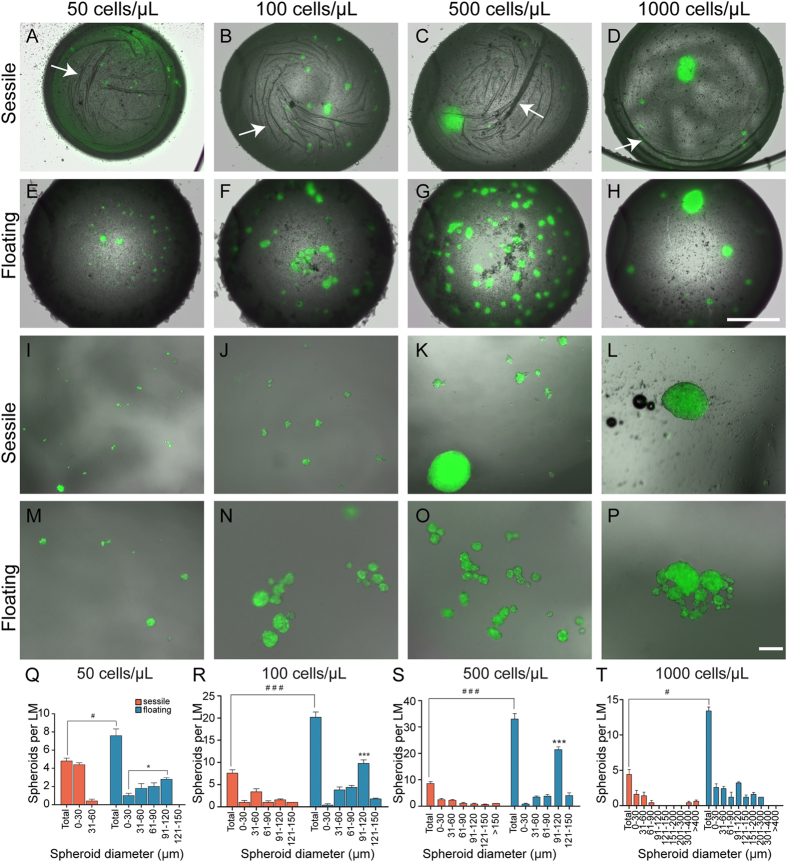
Effect of cell density on formation of spheroids. Spheroid formation inside (**A**–**D**) sessile marbles and (**E–H**) floating marbles at the seeding densities of 50, 100, 500 and 1000 cells/μL. Arrows in (**A**–**D**) show creases in PTFE layer indicating dehydration of marbles. (**I**–**L**) Sessile and (**M**–**P**) floating marbles were broken and spheroids allowed to settle to bottom of the well; panels show spheroids from a single broken marble at each density of 50, 100, 500 and 1000 cells/μL. Scale bar in (**A**–**H**) is 500 μm; (**I**–**P**) is 200 μm. (**Q**–**T**) Quantification of spheroid size. The total number of spheroids and the number of spheroids within the indicated diameter ranges in sessile (orange) and floating (blue) marbles seeded with (**Q**) 50 cells/μL, (**R**) 100 cells/μL, (**S**) 500 cells/μL and (**T**) 1000 cells/μL. Floating marbles produced significantly more spheroids than sessile marbles at all seeding densities. (^#^p < 0.05 and ^###^p < 0.001, n = 10 liquid marbles). In floating LMs, there were significantly more spheroids in the diameter range 91–120 μm compared to other sizes (*p < 0.001 and ***p < 0.001 n = 10 liquid marbles, repeated three times). Data were analysed using one-way ANOVA followed by post hoc Bonferroni’s test. All experiments were repeated three times. Bars represent the mean; error bars represent the standard error of the mean.

We investigated the cell seeding density inside the LM and found that it plays an important role in the yield and uniformity of the spheroids. The 10 μL volume LM containing four different cell seeding densities (50, 100, 500 and 1000 cells/μL) were investigated in sessile and floating conditions. The sessile LMs at seeding densities of 50 and 100 cells/μL produced numerous small spheroids of cells (Fig. 3A,B), while at 500 and 1000 cells/μL the sessile LMs produced a single large mass with several smaller spheroids (Fig. 3C,D). When viewed with bright field, it was apparent that the sessile marbles underwent evaporation as wrinkles in the silicon coating were clearly visible (Fig. 3A–D). In contrast, the floating LMs produced spheroids that were distinctly different to those produced in sessile LMs. At seeding densities of 50–500 cells/μL numerous spheroids were produced (Fig. 3E–G) while at 1000 cells/μL a single large mass was produced along with smaller spheroids (Fig. 3H). There was no apparent shrinkage of the hydrophobic shell indicating that evaporation was reduced compared to the sessile LMs. Further examination of the effect of evaporation revealed that after 48 h the shrunken sessile 10 μL LMs often collapsed whereas floating 10 μL LMs maintained their surface integrity for up to 72 h.

We next broke the marbles to release the contents into the wells of the 96-well plates to better observe the spheroids that had been produced. The sessile LMs with seeding densities of 50–500 cells/μL produced numerous small spheroids, with a larger mass being produced at 500 and 1000 cells/μL (Fig. 3I–L). The floating LMs at seeding densities of 50–500 cells/μL produced more numerous and larger spheroids compared to those formed in sessile LMs (Fig. 3M–O), and at 1000 cells/μL produced larger spheroids that were more spherical than that produced in sessile LMs (Fig. 3P vs 3L). We measured the diameters of the spheroids that were produced at the different seeding densities in both the sessile and floating LMs. At all seeding densities, the floating LMs produced more spheroids and produced larger spheroids (Fig. 3Q–T). One-way ANOVA analyses indicated that cell densities of 100 and 500 cells/μL produced significantly more spheroids of the size range 91–120 μm (Fig. 3R,S, p < 0.05 and p < 0.001). Floating LMs at density of 500 cells/μL generated high yields of spheroids with an average of 33 ± 2.1 spheroids of which 49.7% had a diameter in the range of 90–120 μm (Fig. 3S). Floating LMs with a seeding density of 1000 cells/μL also produced several spheroids with diameters of more than 200 μm (Fig. 3T). In contrast, sessile marbles were not able to produce such high yields. The larger spheroids that were produced by sessile LMs resembled tissue sedimentation as they had irregular shapes and appeared to suffer from cell degradation at the edges (not shown). This was highlighted by the very large irregular shaped sedimentation (>400 μm) that was observed at seeding density of 1000 cells/μL.

### Assessment of OEC migration out of spheroids

**Figure 4:**
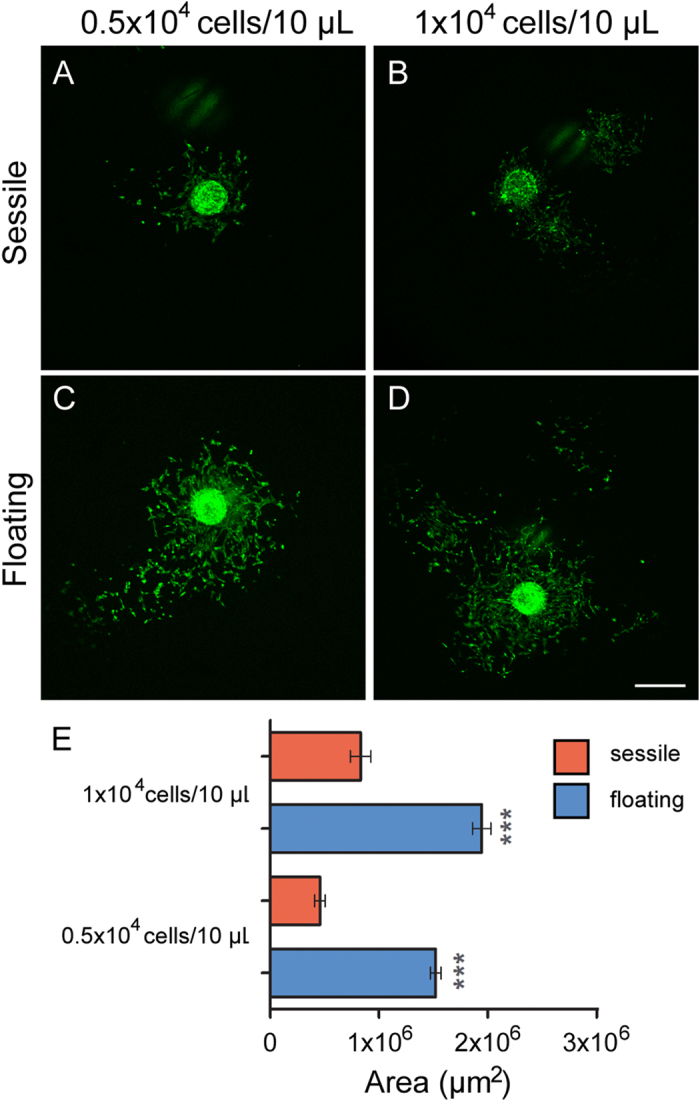
Migration of cells out of spheroids grown in sessile and floating marbles. Sessile and floating marbles were seeded at 500 cells/μL and 1000 cells/μL and cultured for 48 h; spheroids were then removed from marbles, plated out and cultured for 24 h. Cells migrated further from spheroids grown in (**C**,**D**) floating marbles than (**A**,**B**) sessile marbles at both cell seeding densities. Scale bar is 500 μm. (**E**) Measurement of migration of cells from spheroids grown in sessile and floating marbles; the area over which the cells had migrated was measured at 24 h after plating. Cells from spheroids grown in floating marbles migrated significantly further than cells from sessile spheroids (***p < 0.001; n = 30 spheroids). Data were analysed using one-way ANOVA followed by post hoc Bonferroni’s test. Bars represent the mean; error bars represent the standard error of the mean.

The morphology and size of the spheroids within floating LMs indicated that the growth and interactions of the OECs were more favourable than the conditions within sessile LMs. It is well-established that cells migrate out of spheres with higher migration rates when cells are healthy and robust. To assess the migration of cells out of spheroids, we used grew spheroids in 10 μL sessile and floating LMs with seeding densities of 500 and 1000 cells/μL and then broke the marbles and plated the spheroids in wells of a 96-well plate to allow the OECs to migrate out of the spheroids onto the surrounding surface (Fig. 4A–D). Spheroids with diameters between 90–120 μm at t = 0 h were selected and the region in which the cells had migrated at t = 24 h was measured. One-way ANOVA analyses showed that there was significantly more migration (p < 0.001) of OECs from spheroids grown in floating LMs at both 500 and 1000 cells/μL than from spheroids grown in sessile LMs (Fig. 4E), with OECs from floating spheroids migrating out over an area 2–3 times larger than OECs from sessile spheroids.

### Morphological analysis of spheroids

**Figure 5:**
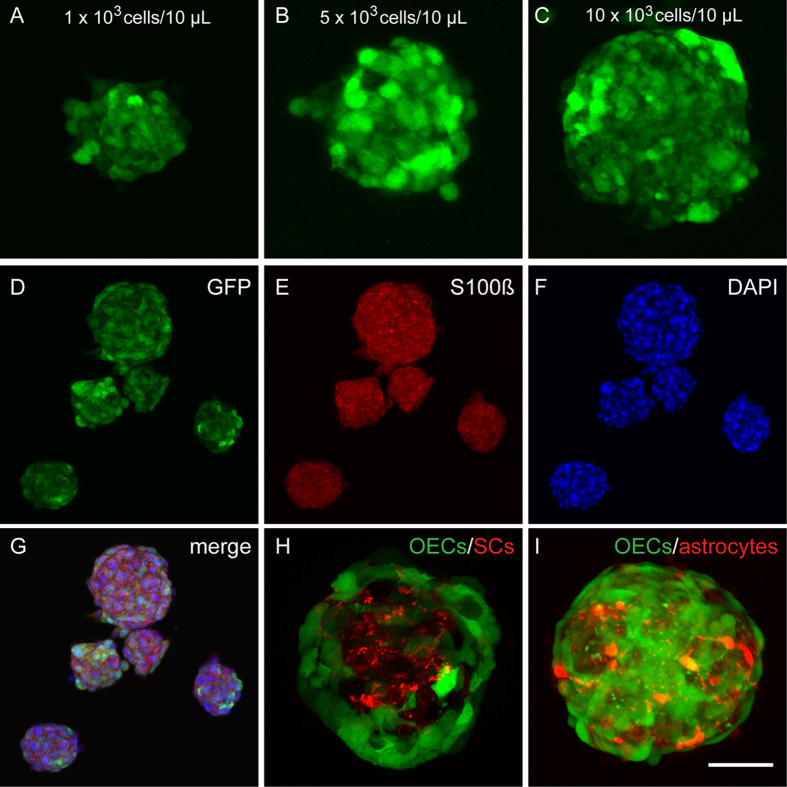
Cells formed spheroids with extensive cell-cell contact. Panels show confocal images of spheroids isolated from LMs. (**A**–**C**) LMs with different cell densities produced different sized spheroids which all had extensive cell-cell contacts (**A**) 60 μm diameter spheroid, (**B**) 100 μm, (**C**) 150 μm. (**D**–**G**) Immunostaining of spheroids from floating LMs grown for 24 h at a seeding density of 5 × 10^3^ cells/10 μL: (**D**) GFP-expressing OECs, (**E**) s100β immunostaining, (**F)** DAPI and (**G**) merged image. (**H**,**I**) Co-culture of OECs with Schwann cells and with astrocytes. Marbles were seeded with the two different cell types at a ratio of 1:1 with an overall seeding density of 500 cells/μL and cultured for 48 h. Spheroid co-cultures of (**H**) OECs (green) with Schwann cells (red) and (**I)** OECs (green) with astrocytes (red). Scale bar is 50 μm in (**A**–**C**); 200 μm in (**D**–**G**); 60 μm in (**H**,**I**).

We used confocal microscopy to visualise the structure and morphology of the OECs within the spheroids. The OECs express GFP with some cells expressing higher levels than others depending on their individual growth conditions. Spheroids of different sizes from floating LMs were imaged by confocal microscopy. OECs within all sizes of spheroids clearly showed extensive cell-cell interactions with processes extending in all directions indicating robust growth and cellular interactions (Fig. 5A–C). We next performed immunocytochemistry with antibodies against s100β which is a standard markers of OECs. The immunostaining revealed uniform expression of S100β throughout the spheroid including the internal central region of the spheroid which indicates that OECs throughout the spheroid were healthy (Fig. 5D–G).

### Generation of co-culture spheroids

A better understanding of the behaviour and interaction of OECs with other glia cells can be elucidated more efficiently by using a 3D co-culture model with a mix of different cell types. To test the applicability of using floating LMs for co-culturing different glial cells, we seeded the LMs with two different combinations of cells: (1) OECs+Schwann cells, and (2) OECs+astrocytes at a ratio of 1:1 with a total cell density of 500 cells/μL. The co-cultured spheroids were fixed and imaged after 48 h. Confocal imaging showed that co-cultured cells developed spheroids with specific cellular organization and localization (Fig. 5H,I). When co-cultured with Schwann cells, the OECs localized to the outer layer of the spheroid and enveloped the Schwann cells which populated only in the inner core of the spheroid (Fig. 5H). When co-cultured with astrocytes, the OECs were also predominantly restricted to the exterior of the spheroid although there was clearly more cell-cell intermingling with the astrocytes than had occurred with OECs and Schwann cells (Fig. 5I).

## Discussion

The floating liquid marble platform that we describe here provides exceptional properties for culturing cells in three dimensions. With this micro-bioreactor, we have shown that OECs can freely associate to rapidly form numerous spheroids that provide robust growth and proliferation of the cells. Importantly, we demonstrate that co-culturing of different cell types within this floating platform reveals unique characteristics of cells that have not previously been reported. We envisage that floating LMs can be used to interrogate the complex cell interactions that occur as cells form 3D structures which is essential for further improving therapeutic use of cells in transplantation therapies.

Although a number of studies have described the utility of various 3D culture systems for OECs^13^,^25^ the important challenge is to create 3D heterogeneous tissue constructs without exogenous scaffolds. Compared with other 3D tissue models, the generation of spheroids using floating LMs do not require any solid supports such as hydrogels, scaffolds or matrices to obtain a 3D arrangement. This is an important advance as degradable or non-degradable biomaterials can interfere with the cell physiology and potentially alter cell function. The use of the hydrophobic PTFE powder to encapsulate the marble has several advantages. While the coating may appear entire when viewed by eye, at high magnification the coating is not uniform which confers useful properties to the marbles. The distribution of particle coating has been previously described using environmental scanning electron microscopy which indicated that the particle coating is not uniform or continuous and that air is likely trapped within water clearings on the surface coating which promotes Leidenfrost-like behaviour^22^ which is likely to aid fluid flow within the marble and thus promote cell-cell interactions.

Our technique is a further advancement on the previous well-known hanging drop model. Although the hanging drop method has been validated and widely used^26^, this method is based on gravity-induced spheroid formation that does not produce multiple reproducibly-sized spheroids. When we examined the formation of the tissue masses in sessile LMs, we found that the morphology indicated poor cell growth likely as a result of excessive sedimentation of cells, particularly in the very large masses of tissues. In contrast, in floating LMs the cells were able to freely associate and formed 2–3 times more spheroids, with the spheroids being considerably larger. While floating LMs did produce spheroids up to 300 μm in diameter, these spheroids appeared healthy. The superior migration of cells out of the floating LM spheroids confirmed that the floating LMs produced a more favourable environment for cell growth compared to sessile LMs. Sedimentation of cells is likely to result in cells forming near-neighbour relationships that would not normally occur. In contrast, the floating LM is presumed to be an environment that permits cells to freely associate is likely to result in cells forming 3D structures that better reflect the *in vivo* context. By floating the LM on a liquid bath, the buoyancy maintains liquid flow within the marble and therefore minimises the effect of gravity that forces the cells to aggregate at the bottom of the LM. Thus floating the LMs is crucial to the utility of this micro-bioreactor.

In the context of stability, a floating LM is relatively robust and behaves like a soft solid structure that can resist small physical impacts during handling. The robustness and easy handling are clear advantages over hanging drops as it can be difficult to maintain the physical integrity of the hanging drop and its contents. The limitation of other scaffold-free methods is discussed in (Table 1).

These techniques display impact on culture uniformity and are generally time consuming. Moreover, all techniques currently available can be laborious and require specialized and expensive culture equipment. Another key advantage of LM is the convenient and real-time observation of the cells inside this bioreactor using conventional microscopy. The fine coating of hydrophobic powders with particle size of 1 μm allows optical access to the content of the marble. Therefore, this protocol is well suited to studies of the assembly of cells with different fluorescent labels by using time-lapse microscopy. In comparison, the hanging drop method is not suitable for live cell imaging and thus the formation of the spheroids cannot be easily tracked over time. We were able to culture cells in floating LMs for up to 3 days and while longer culturing could be possible, the exhaustion of the nutrients necessitates replacement of the medium. Extended culturing can be achieved by passaging the spheroids within the LMs. The LMs can be broken, the cell spheroids collected and resuspended in new medium with new LMs subsequently formed.

We determined that the optimal volume of the floating LMs was 10 μL for our assays. The 10 μL floating LM was found to be more robust and exhibited lower evaporation rate. A sustainable spherical shape and regulation of evaporation rate is crucial for development of identical sized multiple spheroids. We reasoned that disruption of LM spherical structure is due to the gravitational effect on the marbles and that the evaporation rate can be presumed to be relative to the surface area/volume ratio. We have previously calculated that the LMs at 10, 20, 50 μL have Bond numbers of 0.26, 0.41 and 0.77 respectively^24^. The smaller the Bond number, the more spherical it is due to dominance of surface tension forces over gravitational forces. Although a sessile 10 μL LM sustains a spherical shape, it will have a higher evaporation rate because of its smaller bond number with higher surface/volume ratio. Thus we concluded that the optimal size of the marbles to maintain the spherical shape was 10 μL and that by floating the LMs the evaporation rate is reduced which thereby maintains the appropriate concentrations of nutrients within the media necessary for optimal cell growth.

Optimizing the LM size has important implications for other assays such as drug screening. In particular, large scale drug screening can be expensive due to the volume of reagents and growth factors that are needed, as well as the requirement for using larger amounts of what are often limited quantities of novel candidate drugs. By having a maximum volume of 10 μL our protocol cost saving because the microfluidic scale of the LM limits the amount of reagents such as growth factors or test compounds needed for the assays. Thus, our floating LM 3D culture assays satisfy several key requirements for large-scale drug screening such as speed, simplicity, reproducibility and cost effectiveness. The ease of production of the LMs may be translated to generate an automated digital microfluidics system. This could be achieved by using robotic liquid handling to dispense precise droplet volumes to enable high throughput production of LMs. This potentially serves as a powerful tool for automated cell spheroid culturing for applications such as drug screening.

Due to their various potential regenerative properties, combinations of OECs and Schwann cells have been transplanted into the injured spinal cord where they have shown effectiveness in promoting neural repair^27^,^28^,^29^. However, OECs and Schwann cells exhibit differences in survival, migration and proliferation when transplanted into neural tissue^30^,^31^,^32^ and they interact differently with astrocytes^33^. When we examined the interactions of OECs with Schwann cells and with astrocytes in floating LMs, the OECs became localised to the exterior of the spheroids and ensheathed both the Schwann cells and astrocytes. This behaviour of OECs has not been previously observed and thus the floating LMs create a unique environment in which novel cell behaviours can be observed. Within their endogenous environment of the olfactory system, OECs of the lamina propria ensheathe bundles of axons and are largely restricted to the exterior of the nerve fascicles, with their processes penetrating into the deeper regions of the fascicle^34^. Thus the co-culture LM model has enabled the OECs to freely associate with other cell types and replicate their normal behaviour of surrounding other cells. The use of floating LMs therefore appears to be particularly favourable for interrogating the interaction of co-cultured cells.

In summary, floating LMs are a simple, fast and low-cost method for generating multiple uniform spheroids without specialized equipment. The floating LMs permit cells to freely associate and form complex three-dimensional structures with high viability and reproducibility. Thus, the use of floating LM is a paradigm-shifting multipurpose tool for performing basic and complex experiments for the understanding the use of glia for neural repair therapies, with the potential for use in large scale drug screening.

## Methods

### Cell culture

The GFP-expressing immortalized mouse OECs were previously generated^35^. Cells were cultured in DMEM/F12 (Life Technologies) supplemented with 10% FBS (vol/vol), 2 μM forskolin (Sigma), 20 μg ml^−1^ pituitary extract (Gibco), 10 ng ml^−1^ FGF-2 (PeproTech), 10 ng ml^−1^ EGF (PeproTech) and 0.5% (vol/vol) gentamicin (Life Technologies). The sub-confluent OECs in T25 were washed twice with HBSS (Life Technologies) and detached with TrypLE Express (Life Technologies) for 5 min at 37 °C. The enzymatic reaction was stop by addition of 2 mL 10% FBS media and the solution centrifuged at 1000 rpm for 5 min. The OEC culture medium was changed every 2 days. Primary cultures of Schwann cells and astrocytes were prepared from S100ß-DsRed transgenic mice^36^. Postnatal day four (P4) mouse pups were sacrificed by decapitation following guidelines established by National Health and Medical Research Council of Australia (NHMRC); protocols were approved by animal ethics committee of Griffith University (permit number: ESK/05/12/AEC). For Schwann cells, dorsal root ganglia from the spinal cord were dissected and explants plated in 24-well plates previously coated with Matrigel (BD Bioscience 1:10). Astrocytes were prepared from cerebral cortices, meninges membranes were removed and the middle neocortical portion of the cerebral cortex isolated and placed in a petri dish containing 2 mL of TrypLE Express (Life Technologies) solution for 15 min at 37 °C. This reaction was stopped by addition of 2 mL 10% FBS and the solution centrifuged at 200 g for 7 min. The tissue was resuspended in complete medium (10% FBS) and incubated in 5% CO_2_ at 37 °C after plating on a 24-well Matrigel coated plate. Schwann cells and astrocytes were cultured in DMEM (l-glutamine) supplemented with 10% (vol/vol) FBS, 0.1% gentamicin, 0.5% glutamax (Life Technologies), 0.2% G5 supplement (Life Technologies). Medium was changed every 3 days and at 7^th^ day the cells are ready for experiment. All cell types were cultured in humidified atmosphere with 5% CO_2_ in air at 37 °C.

### Generation of spheroids in Liquid marble

To create the liquid marbles, a polytetrafluoroethylene (PTFE) (Sigma-Aldrich, product number 430935) powder bed with particle sizes of 1 μm was prepared inside a 6-well plate. A micropipette was then used to dispense the required volume of OEC medium containing a pre-determined number of cells (50, 100, 500 and 1000 cells/μL) on the powder bed with the preferred volume which ranged between 10 to 50 μL. For co-culture spheroids, Schwann cells and astrocytes were prepared at the 1:1 ratio at the cell density of 500 cells/μL in 10 μL volumes. The droplets containing cells were dispensed on the PTFE powder bed, followed by gentle shaking in a circular motion for 3 min. All cell types were cultured in humidified atmosphere with 5% CO^2^ in air at 37 °C.

### Floating the liquid marble

Droplets coated with PTFE powder were picked by using 1000 μL pipette tips which were cut at the edge to accommodate the diameter of the marble. Then the liquid marbles were placed carefully in wells which did not contain any medium in the 96-well plates to float the marble, 100 μL media was added to the wells without direct contact with the marble. The liquid marble content was then monitored during incubation, using an optical–fluorescent microscope (Olympus IX70), to examine the cell growth and spheroid formation.

### Preparation of spheroids

Floating LMs were broken by puncturing with a needle. Sessile LMs were broken by addition of 100 μL of media directly onto the LM to disperse the hydrophobic coating. Microscopic examination was carried after spheroids settled in the wells. For immunohistochemistry staining, spheroids were obtained from multiple wells by centrifugation for 5 min at 1000 rpm.

### Spheroid migration assay

The spheroids were transferred to a flat bottom 96-well plate under standard cell culture conditions and incubated for 24 hours to evaluating the migration capacity and then imaged with a fluorescence microscope (Olympus IX70) and imaging software (SPOT). The migration out of the spheroid was measured by subtraction of the spheroid radius at t = 0 from the farthest distance of migrated cells.

### Immunocytochemistry

Spheroids were fixed by adding 200 μL of fresh 4% (wt/vol) paraformaldehyde (PFA) (Sigma-Aldrich) to the wells containing spheroids and stored overnight at 4 °C. Spheroids were washed three times with 0.1 M phosphate buffered saline (PBS) and transferred to a 1.5 mL microcentrifuge tube in which the entire immunocytochemistry procedure was performed. The spheroids were blocked in 2% bovine serum albumin (BSA) in PBS with 0.3% Triton X-100 (TX) at room temp (25 °C) for 30 min and then incubated with rabbit-anti S100β (Santa Cruz, 1:500) overnight at 4 °C. After washing 3 times, spheroids were incubated with goat anti-rabbit antibodies conjugated with Alexa 594 (Invitrogen) for 4 h at room temp and then washed and stained with DAPI for 15 min at room temp. Spheroids were washed and stored in PBS for imaging. All solutions are removed by washing 3 times with PBS and centrifugation for 5 min at 1000 rpm.

### Statistical method

Spheroid diameter was analysed by non-paired one way ANOVA with Bonferroni multiple comparison test at each time point. The differences were significant when p < 0.05.

## Additional Information

**How to cite this article**: Vadivelu, R. K. *et al.* Generation of three-dimensional multiple spheroid model of olfactory ensheathing cells using floating liquid marbles. *Sci. Rep.* **5**, 15083; doi: 10.1038/srep15083 (2015).

## Figures and Tables

**Table 1:** Limitation of the techniques for scaffold-free spheroid production.

Techniques	Description	Problems
Pellet Culture	Centrifugal force used to concentrate cells to the bottom of tube to form spheroids	• Irregular shaped single spheroid with necrotic morphology^37^
Spinner Culture	Single cells are prevented from settling by continuous suspension	• Induce shear stress and increase tissue necrosis^38^
Hanging Drop	Gravity enforced in hanging droplets enhances self-assembly to produce spheroids	• Single spheroid^39^ Limitations: not suitable for microscopic tracking, media exchange media or adding drugs^40^
Rotation Wall Vessel	Aggregation of cells to form spheroids using microgravity effect	• Variability in spheroid size^41^
External Force	External force e.g. ultra sound, electric field or magnetic force used to aggregate cells	• Morphological changes^42^
